# The Draft Poor-Law Order

**Published:** 1912-09-28

**Authors:** 


### THE DRAFT POOR-LAW ORDER.

Threatened men proverbially live long; and the proverb is even more applicable to threatened institutions in an old country like this. The fuss and fume of the Poor-Law Commission Report has died away to a considerable extent, superseded by more exciting even if less intrinsically important political issues and intrigues. In the present case a Departmental Committee of the Local Government Board has been considering the management of Poor-Law institutions, and has issued a draft order to regulate their conduct. How far the work of the Poor-Law Commission has been kept in view is not apparent on the surface; at least, the provisions of the draft which affect the status and duties of the medical officers of infirmaries bear evidence of an intimate knowledge of the interior economy, such as only expert officials would be likely to possess. The suggested order, in so far as it concerns the medical officers of infirmaries, was published in last week's issue of The Hospital, but some of the points are worthy of closer consideration, as they modify very materially the hitherto established order of affairs.

To the regulation numbered (1) no reasonable objection can be made; it sets forth with clearness the general principle of the relation between the Guardians and the employed medical officer. The next paragraph prescribes a minimum which in practice is certainly always far exceeded; no conscientious medical officer would want to expend less care upon the children of his infirmary than is here laid down as obligatory. Paragraphs three and four relate chiefly to the compilation of returns which are not arduous; if routine work of this sort is irksome to some men's characters, it is nevertheless necessary to recognise that in a public institution a certain amount of it is unavoidable.

Perhaps the most important single paragraph is the fifth, which gives the medical officer his due authority over the whole of the nursing staff from the superintendent nurse and the matron downwards. The provisions of the eleventh paragraph explain and extend this authority, by making it perfectly clear what his remedy is should he be dissatisfied with the nursing staff, or any member of it, or with the surgical and medical equipment of the institution, or with the arrangements which he can require the master to make in order to deal with any emergency that may arise. These two paragraphs taken together are much more explicit and much more satisfactory than those which existed in the consolidated order hitherto in force. The sixth, seventh, and eighth paragraphs are of less importance; they deal with the functions of the medical officer in respect to mental and infectious cases, and other special duties. The ninth paragraph says that the medical officer must furnish to the medical officer of health for the district such information as the Board of Guardians may direct concerning sickness or deaths within the institution. This puts the medical officer in the hands of the Guardians to some extent; but we cannot see that any hardship is likely to arise, and the provision, if reasonably construed, is quite unexceptionable.

The tenth paragraph seems to us less satisfactory. It requires the medical officer, besides furnishing the Guardians with information about any specified patient (which is quite proper), to furnish also all certificates called for by the patient or by any person acting on his behalf. Presumably this implies that such certificates shall be furnished free of expense, since no permission to charge a fee is hinted at. This seems to us a provision capable of abuse. The object probably is to prevent the medical officer from degenerating into a perquisite-hunter. With that object a certain amount of sympathy can be felt; but this method of attaining it seems to us unjust and unworkable. In cases of accident or of disease questions of compensation frequently arise; certificates are required, and they should be paid for. Insurance companies, employers, and employed often ask for detailed reports in such cases, especially if there is a probability of the dispute being taken into court. There is no reason why they should not pay fees for these luxuries, just as they would to a voluntary hospital officer or to a private practitioner.

